# Proceedings of the Twenty-Seventh Annual Meeting of the Mississippi Valley Dental Association, March 1st, 1871

**Published:** 1871-05

**Authors:** 


### Proceedings of Societies.

### PROCEEDINGS OF THE TWENTY-SEVENTH ANNUAL MEETING OF THE MISSISSIPPI VALLEY DENTAL ASSOCIATION, MARCH 1st, 1871.

Society met in lecture room of the Ohio Dental College. Dr. W. H. Morgan, President, in the chair. Society adjourned after some informal conversation, until afternoon.

WEDNESDAY, 2 P. M.

Minutes of last meeting were read and approved, and the roll called.

Dr. Goddard, moved that absent members of the Committee on Membership be filled. (Dr. Rosson, the only member of that Committee present.) H. A. Smith, and H. McCullum were appointed to fill the vacancy. Dr. Canine, of Louisville, was presented by the Committee on Membership and elected unanimously. On motion, Dr. Wright was appointed to report the proceedings of the society, at a reasonable compensation. A discussion on the subject of the heavy and light mallets followed.

Society adjourned, to meet Thursday morning, March 2nd, at 9 o'clock.

THURSDAY, 9 A. M.

Society met according to adjournment.

President W. H. Morgan in the chair.

Minutes of the last meeting read and approved.

The following names were presented by the Committee of Membership:

Drs. E. Osmond, J. A. Stipp, Jno. Rabe, S. C. Harryman, Chas. Welch, J. R. Wilkings, R. E. Finley, C. S. Case, J. E. Cravens, W. T. Wallace, L. B. Welch.

A motion was made to vote on the whole list, and if one black ball be cast, the names be taken up singly and balloted for. Carried.

A black ball having been cast, the names were taken up singly. Dr. E. Osmond being balloted for, received ten yeas and nine nays. A two-thirds vote being necessary to elect candidates to membership, the name was dropped. The objection raised was for advertising as a steam dentist, and for unprofessional conduct toward other dentists. The rest of the names were then taken up singly, and all unanimously elected.

The subject of heavy or light mallets was then laid aside, and the next subject taken up, "treatment of soft tissues of the mouth."

Society adjourned till 2 o'clock, P. M.

THURSDAY, 2 P. M.

Society met at 2 o'clock, P. M. Dr. W. H. Morgan presiding.

Minutes of last meeting read and approved.

Dr. H. J. McKellops called the societies attention to the death of Dr. Peebles, of St. Louis, and Drs. Goddard, Taft and Rehwinkle, were appointed a committee to draft resolutions suitable to the event. On motion, Dr. Keeley and Dr. McKellops were added to the committee.

Dr. J. Taft gave information of the attempt to amend the Ohio State dental law, and of the failure of this attempt. He urged upon members from other States to work for the establishment of a similar law in their States. He informed the members that our law is at present on a firm basis.

Dr. Taft moved that a committee be appointed to draft resolutions of thanks to Dr. Brook and other legislators, for their efforts in behalf of the dental law—and Drs. Keeley and Smith were appointed on committee.

The next subject for discussion was then taken up, after which the election of officers took place.

*President*—H. A. Smith, Cincinnati.
*Vice-President*—J. F. Canine, Louisville.
*Recording Secretary*—C. M. Wright, Cincinnati.
*Corresponding Secretary*—Merrit Wells, Indianapolis.
*Treasurer*—J. G. Cameron, Cincinnati.

Society adjourned to meet Friday, at 9 o'clock, A. M.

FRIDAY, 9 A. M.

Society met at 10 o'clock. President, Dr. H. A. Smith, in the chair.

Minutes of last meeting read and approved.

Dr. Goddard, moved a re-consideration of the vote taken yesterday, excluding Dr. E. Osmond from membership, claiming that as he voted against his admission yesterday under a misapprehension, he desired to re-consider his vote. The vote was re considered. Dr. Osmond made satisfactory explanation. A vote was then taken, and he was elected a member. Dr Thomas C. Fahnestock, of Cincinnati, was elected to membership.

The following delegates were elected to represent this society in the next American Dental Association, at White Sulphur Springs, Va.

Dr. J. F. Canine, Dr. Merrit Wells, Dr. J. E. Cravens, Dr. L. P. Meredith, Dr. R. A. Mollyneaux, Dr. A. O. Rawls, Dr. J. A Stipp, Dr. E. C. Sloan, Dr. Chas. Welch, Dr. R. E. Finley.

Dr. Morgan, the retiring President, delivered a short address.

Dr. Rehwinkle, offered the resolution that hereafter the incoming and newly elected President, shall deliver an address on the afternoon of the first day of our annual meeting. Carried.

The following Executive Committee was appointed by President Smith, viz: Drs. J. Taft, E. C. Sloan, and W. H. Shadoan.

Committee on Membership—Drs. G. W. Keeley, D. B. Wheeler, and P. G. C. Hunt.

On motion of Dr. Watt, the committee who have in charge the stereotype plates and copy right of this association, were allowed discretionary power to dispose of them for the benefit of the society.

On motion of Dr. Goddard, a vote of thanks was offered Dr. Morgan, the retired President.

The following resolution of thanks to the legislators was presented:

WHEREAS, Dental Surgery has demonstrated its claims to consideration as a branch of one of the learned professions; and

WHEREAS, In the State of Ohio, the profession has until recently been unprotected and unsustained by legal enactments, necessary for the highest attainment of usefulness; therefore

*Resolved*, That the thanks and gratitude of this association are due, and are hereby tendered to Dr. Geo. Brooke, of the Ohio House of Representatives, and to his associate members, for securing and maintaining the law for regulating the practice of dentistry in this State; an example of practical benefit to the profession and the public, that we would respectfully and earnestly recommend to sister States.

G. W. KEELEY,<br>H. A. SMITH. } *Com.*

Adopted.

Dr. C. M. Wright read a paper on artificial dentistry.

Society adjourned.

